# Intra-socket application of medical-grade honey after tooth extraction attenuates inflammation and promotes healing in cats

**DOI:** 10.1177/1098612X221125772

**Published:** 2022-10-31

**Authors:** Carlos CF Pleeging, Hilde de Rooster, Bas Van Wijk, Frank ADTG Wagener, Niels AJ Cremers

**Affiliations:** 1 Dierenkliniek Hoogveld, Echt, The Netherlands; 2 Small Animal Department, Faculty of Veterinary Medicine, Ghent University, Ghent, Belgium; 3 Department of Dentistry – Orthodontics and Craniofacial Biology, Radboud Institute for Molecular Life Sciences, Radboud University Medical Center, Nijmegen, The Netherlands; 4 Department of Gynecology and Obstetrics, Maastricht University Medical Centre, Maastricht, The Netherlands; 5 Triticum Exploitatie BV, Maastricht, The Netherlands

**Keywords:** Medical-grade honey, inflammation, wound healing, tooth extraction, dental disease

## Abstract

**Objectives:**

Dental diseases are a major problem in cats and often necessitate tooth extraction. Medical-grade honey (MGH) has antimicrobial and wound-healing properties, and therefore the aim of this study was to investigate whether intra-socket application improved healing after tooth extraction. It was postulated that applying MGH would reduce inflammation, improve the viability of the surgical flap and enhance healing following tooth extraction.

**Methods:**

A prospective randomised controlled trial was performed in client-owned cats undergoing bilateral tooth extractions of the same element of the canine or (pre)molar tooth. A split-mouth design was used in which every animal served as its own control. After surgical extraction of the elements, the sockets on one side were filled with an MGH-based ointment (L-Mesitran Soft), whereas the contralateral side received no treatment (control). A mucoperiosteal flap was used on both sides, and simple interrupted monofilament sutures were placed. No antimicrobial drugs were administered. Clinical parameters (inflammation/redness, flap viability and wound healing) were subjectively analysed on days 3 and 7 post-extraction by a veterinarian blinded to the treatment.

**Results:**

Twenty-one cats were included. MGH significantly decreased signs of inflammation (*P* <0.01), improved mucoperiosteal flap viability (*P* <0.01) and promoted wound healing (*P* = 0.01), at both time points. MGH was easy to apply and there were no adverse events.

**Conclusions and relevance:**

Intra-socket application of MGH after tooth extraction positively affects the surgical wound, as it reduces redness, improves flap viability and enhances wound healing. Applying MGH represents a potent adjuvant therapy to support intra-oral wound healing after tooth extraction.

## Introduction

Dental diseases are a major problem in domestic cats, with a prevalence of 70% in adult cats.^1^ These problems vary from minor periodontal disease to more severe pathologies such as periodontitis (PD), resorptive lesions (RL) or chronic gingivitis stomatitis.^1–3^ These can lead to discomfort and pain, and, in severe cases, require extraction of one or more teeth as a definitive treatment.^4^ Tooth extractions inevitably lead to intra-oral wounds that may cause pain and discomfort associated with the surgical trauma. Medical-grade honey (MGH) could alleviate these issues and promote wound healing. In contrast to unprocessed honey types, MGH adheres to strict criteria to guarantee its safety for the patient and efficacy for wound care.^5^ The beneficial properties for wound care are based on two main pillars: antimicrobial and pro-healing activity.^6,7^ The antimicrobial effect of MGH is partly caused by its intrinsically high osmolality and low pH.^6,8^ Moreover, several bactericidal components, such as local H_2_O_2_ generation, methyl syringate, methylglyoxal, polyphenolic compounds and bee defensin-1, contribute to the antimicrobial effect, depending on the type of flower the bee has pollinated.^9–13^ Simultaneously, MGH positively affects cell migration and proliferation, collagen matrix production and chemotaxis, thereby accelerating wound healing.^14–16^ Finally, phenolic constituents of MGH act as antioxidants, scavenging free radicals created by the activated neutrophils and macrophages, thus protecting the wound microenvironment against oxidative damage.^17^ MGH-treated skin wounds have been demonstrated to heal faster than wounds treated with other dressings or conventional treatments.^18–21^

Most studies have investigated the use of MGH in skin wounds; however, experimental studies in animals and recent clinical studies in human patients showed beneficial effects of topical intra-socket use of MGH after tooth extraction.^22–28^ An experimental study in rabbits and one in rats was conducted, whereby honey was applied into the socket after extraction and then surgically closed by suturing.^22,23^ In the control group, the socket was left to fill with a blood clot and was then closed surgically. The rats were euthanased at 7 and 21 days post-extraction, and all rabbits on day 7. The sockets were then evaluated histologically. The intra-socket post-extraction blood clot in the honey-treated sockets in both rats and rabbits showed more fibrous tissue and more bone trabeculae formation than the controls, indicating further progress in the healing phase.^22,23^ Several clinical trials in humans have also studied the effect of honey on post-extraction sockets. However, the treatment protocol and follow-up intervals are quite different in the various studies,^29^ hampering comparison. In one study, fewer inflammatory signs such as redness, oedema and halitosis were found in the honey-treated group. However, wound size was not significantly different between the honey-treated and control groups.^28^ In contrast, another study in humans found a faster reduction in the wound size of open sockets after a single honey treatment directly after tooth extraction.^26^ A human double-blinded clinical trial observed lower pain scores and less painkiller intake after tooth extraction with a single intra-socket application of honey before surgical closure.^27^ No side effects or allergic reactions to MGH were noticed in any of the patients enrolled in these studies.

Studies of intra-socket application of honey after tooth extractions have not yet been performed in cats. Given the positive results observed in other species, we postulated that MGH applied in sockets after tooth extraction in cats would enhance postoperative intra-oral wound healing.

## Materials and methods

### Ethical statement

This work involved the use of non-experimental animals only. The MGH formulation used in this study is a registered product approved for topical and intralesional use in humans and animals. Therefore, approval from an ethics committee was not required; formal confirmation to waive this was obtained from the Central Authority for Scientific Procedures on Animals (Centrale Commissie Dierproeven) in the Netherlands and from the animal welfare officer from the local Animal Experiment Committee (Dier Experimenten Commissie) at Radboud University, Nijmegen, the Netherlands. Informed consent to participate in the study was obtained from all cat owners before inclusion.

### Animals

Client-owned cats with severe dental disease (feline gingivitis stomatitis complex [FGSC], RL and/or PD) requiring bilateral tooth extractions of the same element of the canine or (pre)molar tooth were enrolled in the study. Whenever the cats were aged 7 years or older, routine blood analyses were performed before surgery to rule out underlying diabetes, kidney disease or hyperthyroidism. Cats suffering from any of these diseases were excluded from the study.

### Study design

A prospective randomised controlled trial was performed. A split-mouth design was used in which the elements of the canine or (pre)molar teeth were divided into a left and a right side. The treatment side was selected at random by the ‘flip of a coin’, and the contralateral side functioned as a control.

### Tooth extraction procedure

Anaesthesia administration and the monitoring of all cats were conducted according to the American Association of Feline Practitioners’ anaesthesia guidelines.^30^ All cats received a similar anaesthetic and analgesic protocol. First, they received 5–10 µg/kg dexmedetomidine (Dexdomitor, Orion) and 5 mg/kg ketamine (Ketamidor; Richter Pharma). Laryngeal spasms were prevented by the application of 0.2 ml 2% lidocaine HCl (Eurovet Animal Health) on the arytenoids. After the lidocaine treatment, cats were intubated and general anaesthesia was maintained with isoflurane (Isoflurin; Chemical Ibérica) in 100% oxygen. The cats also received meloxicam (0.1 mg/kg SC [Metacam; Boehringer Ingelheim]). When cats needed extraction of all elements, 0.03–0.06 mg/kg intravenous buprenorphine (Buprecare; Recipharm) was administered. Monitoring included cardiac and respiratory frequency, temperature, pulse oximetry and capnography. A heat blanket maintained body temperature during anaesthesia. A thorough dental inspection was performed under general anaesthesia, followed by dental radiographs to determine which teeth needed extraction. Dental radiographs were taken with intra-oral phosphorus image plates (Dürr Image Plates; Dürr Dental) using the bisecting angle technique according to Niemiec.^31^ The radiographs were subsequently scanned with a digital image plate scanner (CR 7 VET scanner; Dürr Medical). Teeth requiring extraction were documented in the patient’s digital file. The MGH treatment side was selected randomly by the ‘flip of a coin’. The first side to be treated was randomly selected to rule out potential bias. The teeth were surgically extracted after creating a mucoperiosteal flap, according to Reiter and Soltero-Rivera.^32^ When the affected teeth were multi-rooted, the teeth were hemi-sectioned using an air-powered, water-irrigated fissure bur (iM3 GS Deluxe ‘LED’ Dental Unit) to enable the individual extraction of crown-root segments. Then, the same bur was used to remove the alveolar bone on the buccal side of the tooth roots to make extraction easier using a winged dental elevator and root tip luxator. After extraction, sharp edges of alveolar bone were smoothed with the bur, and the sockets were debrided using a surgical curette followed by flushing with distilled water.^32^

### MGH treatment and control

**Figure 1:**
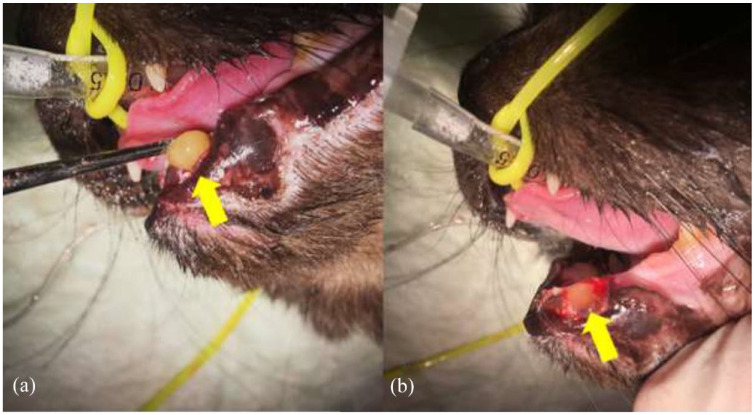
(a) Application of medical-grade honey (MGH) using a spatula after extracting element 304 (yellow arrow). (b) The same cat after the extraction socket of element 304 was filled with MGH (yellow arrow)

**Figure 2:**
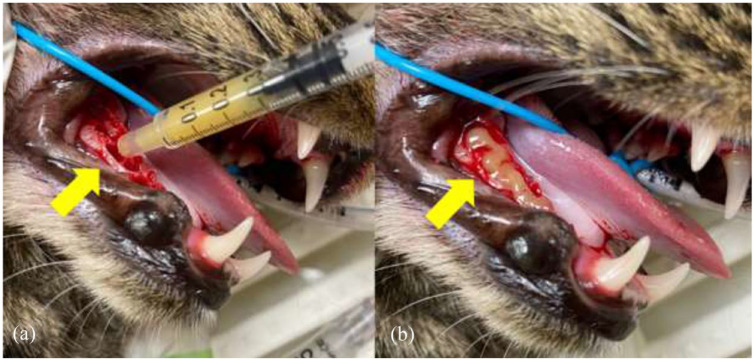
(a) Application of medical-grade honey (MGH) using a syringe in the extraction sockets of 407, 408 and 409. (b) The same cat after the extraction sockets of 407, 408 and 409 had been filled with MGH

**Figure 3:**
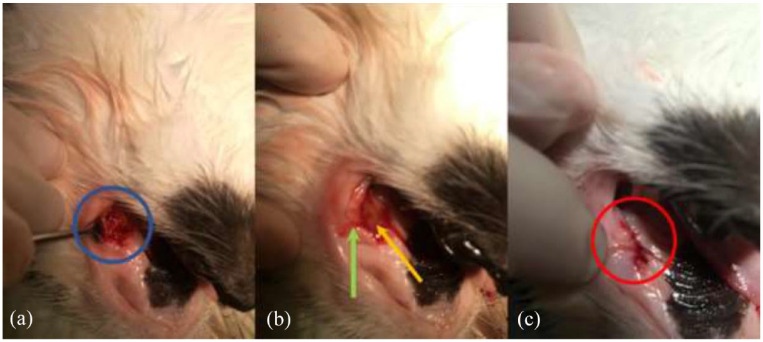
(a) An open socket directly after the extraction of the roots of element 208 (blue circle). The gingival flap is being kept out of the socket using a periosteal elevator. (b) The socket has been filled with medical-grade honey (yellow arrow). The green arrow points towards the elevated mucoperiosteal flap. (c) The mucoperiosteal flap has been sutured over the filled socket with single interrupted sutures in poliglecaprone 5/0 (red circle)

The sockets of the treatment side were completely filled with MGH (L-Mesitran Soft; Triticum Exploitatie), which was applied by syringe or spatula (Figures 1 and 2). Afterwards, the sockets were closed by suturing the mucoperiosteal flap over the defect in the gingiva with single interrupted sutures, applying four throws in each knot, using poliglecaprone 5/0 on a taper point needle (Monocryl; Ethicon). An example of this procedure is presented in Figure 3. On the control side, a blood clot was allowed to form in the socket after flushing, and the mucoperiosteal flap was then sutured as described for the treatment side.

### Aftercare

Postoperatively, pain management consisted of meloxicam (0.05 mg/kg PO q24h [Meloxidyl, Ceva Santé Animale]) for at least 7 days. No antimicrobials were administered. The owners were instructed to provide wet food only until the second recheck on day 7.

### Evaluation of the healing process

Cats were re-presented for follow-up at days 3 and 7 postoperatively. During the mouth inspection in the awake cat, both sides were compared according to the following criteria: redness; the viability of the flap; wound healing; and suture quality. Redness was scored according to the colour of the wound and the flap, and the directly surrounding gingiva were evaluated for this parameter. Flap viability evaluated the mucoperiosteal flap for perfusion and signs of necrosis. For wound healing, the sutured wound was scored for retraction, oedema and discharge at the wound margins. Finally, to evaluate suture quality, the sutures were checked to see if they were still in place and if the knots remained tight. Another veterinarian made a left/right comparison. The observations were later scored as (+) if the treatment side scored better, (0) if they scored equally and (–) if the treatment side scored worse when compared with the control side.

### Statistics

Because the observations are paired, the sign test was used; 0 values were not considered for the measurement of *P* values.^33,34^ Subsequently, more stringent testing was performed where 0 values counted as negative results. The tables of Dixon and Mood were used to calculate the *P* value.^33^ Results were considered significantly different when the *P* value was <0.05.

## Results

### Patient population and inclusion

Twenty-one cats were included in the study over 15 months (Table 1). Information regarding breed, sex, age and aetiology was collected. The included cats showed RL (n = 8) of the teeth, PD (n = 6) and/or feline chronic gingivitis stomatitis complex (n = 8). They required tooth extractions of bilateral maxillary (pre)molars and/or bilateral mandibular (pre)molars and/or canine teeth.

**Table 1:** Overview of included cases

Cat	Breed	Sex	Age (years)	Aetiology	Compared teeth	Treatment side
1	Persian	F	5	RL	307/407	R
2	Ragdoll	M	4	RL	107/207	R
3	Maine Coon	F	11	RL	307/407	L
4	DS	F	8	RL	107/207	L
5	Ragdoll	F	5	RL	307/407	R
6	DS	M	5	RL	107/207	R
7	BS	F	5	RL	307/407	L
8	DS	M	8	RL, PD	104/204, 107/207, 307/407	R
9	DS	M	12	PD	108/208	R
10	BS	F	17	PD	104/204	L
11	Maine Coon	F	2	PD	108/208, 309/409	R
12	DS	M	8	PD	108/208	R
13	DS	F	9	PD	104/204	L
14	Maine Coon	F	1	FGSC	107/207, 108/208, 308/408, 309/409	L
15	Maine Coon	M	2	FGSC	107/207, 108/208, 308/408, 309/409	L
16	Siamese	M	1	FGSC	107/207, 108/208, 308/408, 309/409	R
17	Siamese	M	1	FGSC	107/207, 108/208, 308/408, 309/409	L
18	Maine Coon	M	2	FGSC	107/207, 108/208, 308/408, 309/409	R
19	Ragdoll	M	3	FGSC	107/207, 108/208, 308/408, 309/409	L
20	DS	M	3	FGSC	107/207, 108/208, 308/408, 309/409	R
21	BS	F	2	FGSC	107/207, 108/208, 307/407, 308/408, 309/409	L

Cases are sorted by aetiology, not chronologically

F = female; RL = resorptive lesions; R = right; M = male; L = left; DS = domestic shorthair; BS = British Shorthair; PD = periodontitis; FGSC = feline gingivitis stomatitis complex

### Wound scoring

On days 3 and 7 post-extraction, a left/right comparison was made, and scores were given for the different parameters. If the treatment side scored better than the control side, a ‘+’ was given, if both sides scored equally, a ‘0’ was given, and if the treatment side scored worse, a ‘–’ was given (Table 2).

**Table 2:** Wound scoring at days 3 and 7 post-extraction

Patient	3 days post-extraction				7 days post-extraction			
	Redness	Flap viability	Sutures	Healing	Redness	Flap viability	Sutures	Healing
1	+	+	0	+	0	0	0	0
2	+	−	−	0	+	0	0	0
3	0	0	0	0	0	0	0	0
4	0	0	0	0	0	0	0	0
5	+	+	0	+	NA	NA	NA	NA
6	+	+	0	+	+	0	−	0
7	+	+	0	+	+	+	0	0
8	+	+	0	+	0	+	0	+
9	+	+	0	0	+	+	0	+
10	+	+	0	+	0	0	0	0
11	+	+	0	+	+	+	0	+
12	0	0	0	0	0	0	0	0
13	+	+	0	+	+	+	0	0
14	+	+	0	+	+	+	0	+
15	+	+	0	+	+	0	−	+
16	+	0	0	+	0	0	0	+
17	+	+	0	−	+	+	0	+
18	+	+	0	+	+	+	−	+
19	0	0	0	0	0	0	0	0
20	+	+	0	+	+	+	0	+
21	+	+	0	0	+	0	0	0

NA = not available

### Redness

**Figure 4:**
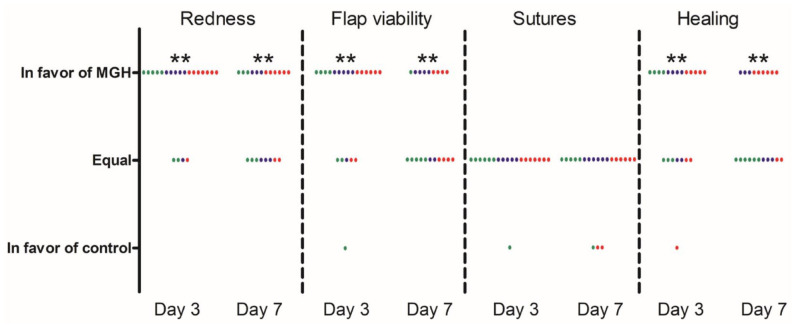
Scoring of the different wound parameters, with the *P* value calculated with the sign test. Green dots represent the cases of resorptive lesions, blue dots the cases of periodontitis and red dots the cases of feline gingivitis stomatitis complex. **P <0.01

**Figure 5:**
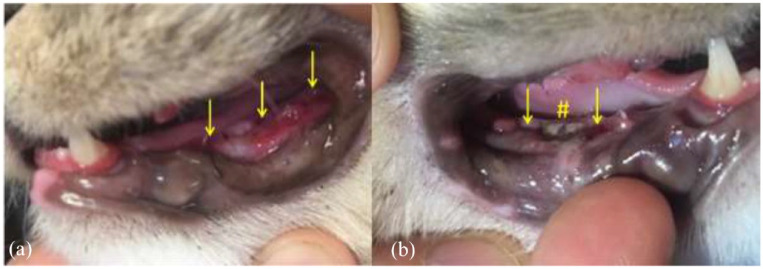
Representative example: Maine Coon treated for feline gingivitis stomatitis complex at the check-up 3 days postoperatively. (a) The control side. The wound and mucoperiosteal flap show more redness (yellow arrows) compared with the treatment side (b) (yellow arrows). There is a small amount of food debris covering the suture (#)

Redness was evaluated as a sign of inflammation. On days 3 and 7, redness after MGH treatment never scored worse than the control treatment (Table 3, Figure 4). The *P* values on days 3 and 7 were <0.005 and <0.01, respectively. Even with the more stringent test, where the 0 values were counted as negative results, the *P* value on day 3 was still <0.005 (Table 4). However, on day 7, the results were no longer statistically significant with the more stringent test (*P* >0.05). Figure 5 shows a representative case where the MGH-treated side had less redness 3 days post-extraction.

**Table 3:** Summary of the scoring of the different wound parameters, with the *P* value calculated with the sign test

	3 days post-extraction				7 days post-extraction			
	Redness	Flap viability	Sutures	Healing	Redness	Flap viability	Sutures	Healing
+	17	15	0	13	12	9	0	9
0	4	5	20	7	8	11	17	11
−	0	1	1	1	0	0	3	0
*P* value	0.005	0.005	−	0.01	0.01	0.01	−	0.01

**Table 4:** *P* values obtained with the more stringent test whereby the 0 values are counted as negative results

	3 days post-extraction				7 days post-extraction			
	Redness	Flap viability	Sutures	Healing	Redness	Flap viability	Sutures	Healing
+	17	15	0	13	12	9	0	9
0 and –	4	6	21	8	8	11	20	11
*P* value	0.005	0.025	−	−	−	−	−	−

### Viability of the surgical flap

**Figure 6:**
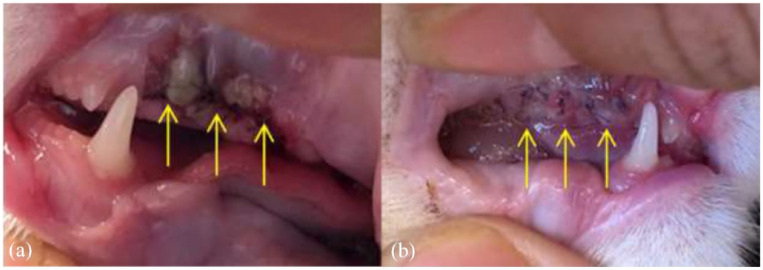
Representative example: British Shorthair cat treated for feline gingivitis stomatitis complex at the check-up 3 days postoperatively. (a) The control side, where the mucoperiosteal flap shows signs of dehiscence and necrosis (yellow arrows). (b) The treatment side, where the mucoperiosteal flap is viable and shows no sign of dehiscence (yellow arrows)

The mucoperiosteal flap was evaluated for viability. Vital signs were good perfusion of the flap and the absence of signs of necrosis. Figure 6 shows a representative case. The *P* values on days 3 and 7 were <0.005 and <0.01, respectively (Table 3, Figure 4). For the more stringent test, the viability was still significantly better for the honey-treated side (*P* <0.025) 3 days post-extraction. However, the results were not significantly different on day 7 (*P* >0.05).

### Sutures

The sutures were compared to evaluate whether the MGH had possible adverse effects on the suture material. In very few cases, the MGH-treated side scored worse than the control side on days 3 or 7 (Table 2). However, these results were not statistically significant (*P* >0.05).

### Healing

**Figure 7:**
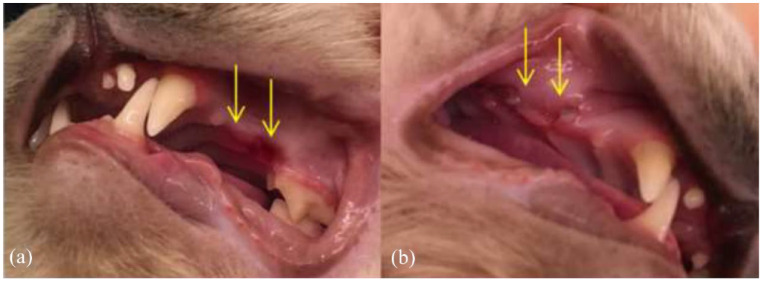
Representative example: Ragdoll treated for resorptive lesions at the check-up 7 days postoperatively. (a) The control side. The wound still has redness and is not entirely healed (yellow arrows). (b) The treatment side is less red, and the wound margins are healed together (yellow arrows)

With regard to healing, the sutured wound was scored on days 3 and 7. The honey-treated group scored better than the control group (*P* <0.01) at both time points (Table 3, Figure 4). However, following the more stringent test, statistical significance was lost (*P* >0.05) on both days (Table 4). Figure 7 shows a representative example.

## Discussion

This study revealed that on both days 3 and 7 postoperatively, MGH treatment significantly reduced redness and significantly improved the mucoperiosteal flap viability and wound healing. Even with the more stringent statistical test, differences in redness and flap viability on day 3 remained significant. The application of MGH was simple and was not time-consuming. No adverse effects of MGH treatment were observed.

A randomised split-mouth design study was performed in which the animals served as their own control, removing inter-animal variations. Days 3 and 7 were selected to offer a window to investigate any possible activity of the MGH. On day 3, there is still inflammation, while on day 7 collagenous union is established in the wound space.^35^ How long healing will take may depend on the aetiology and severity of the extraction wounds. Smaller wounds after single extractions, as is often the case in RL and PD, may heal faster than multiple extractions that lead to larger wounds, as in the case of FGSC. The severity of inflammation may also differ between the diseases. For example, FGSC may be worse at the onset of the extraction and thus at the start of treatment. Despite having included multiple diseases in our study, the statistical significance was still strong. However, it seems that the aetiology influenced the distribution over the different parameters and time points. For example, the aetiologies were equally distributed in terms of the redness at both time points, but flap viability and healing seemed to be more prominently improved in FGSC cats on day 7 vs cats with RL or PD. A larger and/or more inflamed wound at onset may offer a bigger window on day 7 for the MGH to demonstrate its positive effect, as the smaller wounds caused by single extractions might have been almost closed at that time.

This is the first study where the effects of intra-socket application of MGH before surgical closure were clinically evaluated in cats. The observed clinical improvement can be explained by the effects of different molecules in honey that act on a plethora of cellular and molecular mechanisms.^29^ In the first (inflammatory) stage of wound healing, honey stimulates the release of proinflammatory cytokines by monocytes (eg, tumour necrosis factor-alpha, interleukin [IL]-1beta and IL-6) by activation of Toll-like receptor-4.^14–16^ This initial reaction is critical for wound healing. However, after this first inflammatory reaction, honey subsequently suppresses the production of these proinflammatory cytokines by downregulating nuclear factor-kappaB and mitogen-activated protein kinase pathways, and therefore helps in the resolution of inflammation. Also, the formation of reactive oxygen species is reduced by honey.^16,17^ Moreover, the activation of nuclear factor erythroid 2-related factor-2 (NRF2)-target genes, including *HMOX1*, *PRDX*, *SOD*, *TXNRD* and *CAT*, is known to mediate an anti-inflammatory and antioxidative response.^10,16,17,36–40^

Two studies similar to ours, one in rabbits and one in rats, were conducted in which honey was applied into the socket before suturing the intra-oral wound.^22,23^ However, in both studies, the animals were euthanased (rabbits on day 7, and rats on days 7 and 21) and the extraction sockets were evaluated histologically. In both species, histologically, the extraction socket showed more bone-tissue formation in the honey-treated sockets than in non-treated sockets, indicating that the healing process was faster in the honey-treated sockets vs non-treated sockets. The histological observation of enhanced bone formation may be an exciting outcome for human implantology, and this aspect warrants further investigation. In contrast to our study, clinical outcomes, such as redness, flap viability, sutures and healing, were not evaluated in the previous studies.

In human patients, a single intra-socket application of honey following suturing of the mucoperiosteal flap has been assessed in a randomised split-mouth design study.^27^ Contralateral extractions were performed 2 weeks apart, to exclude possible interference. In our study, leakage from the MGH-treated sockets to the opposite mucoperiosteal flaps could not be ruled out, which might have led to an underestimation of the findings. In humans, honey reduced pain scores and the patients needed fewer painkillers in the postoperative period.^27^ Unfortunately, pain is harder to assess in cats than in humans, and therefore a pain score was not included in our study. As inflammation is often accompanied by pain, the decreased redness caused by the potent anti-inflammatory effects of honey may also have resulted in reduced pain in the cats. As pain assessment by the Feline Grimace Scale becomes more common in cats,^41^ this could be a helpful tool in future studies to investigate the effect of MGH on pain in cats. Fascinatingly, some flavonoids that are naturally present in honey show antinociceptive and analgesic effects.^42^ Mu-receptor antagonists could experimentally reverse these effects, suggesting that these flavonoids act like opioid analgesic drugs.^42^ MGH could reduce pain when used in other oral conditions in humans such as mucositis and tonsillectomy wounds, as reported in two meta-analyses.^43,44^

To investigate the underlying mechanism of how MGH improves intra-oral wound healing in more detail, future research may include additional possible objective outcome measures, such as inflammatory markers (systemically in the blood or locally in potential exudate), alveolar bone formation and microbiological colonisation. As these parameters are harder to evaluate, requiring extra blood sampling, CT and/or sedation, and these procedures result in increased discomfort, such investigations would require prior ethical approval. Moreover, despite the strongly significant findings in this study, the sample size was limited. Larger, multicentre studies are encouraged to validate the findings and to expand to other animal species.

## Conclusions

Following tooth extraction, the intra-socket application of MGH before primary closure decreased redness, improved flap viability and enhanced wound healing. MGH was easy to apply, and no adverse events were experienced. MGH represents a valuable aid in treating wounds caused by tooth extraction in cats and can be recommended for application in cats and possibly other species.
